# Cell-Penetrating Peptides: Possibilities and Challenges for Drug Delivery *in Vitro* and *in Vivo*

**DOI:** 10.3390/molecules200713313

**Published:** 2015-07-22

**Authors:** Tore Skotland, Tore Geir Iversen, Maria Lyngaas Torgersen, Kirsten Sandvig

**Affiliations:** 1 Centre for Cancer Biomedicine, Faculty of Medicine, University of Oslo, 0379 Oslo, Norway; E-Mails: Tore-Geir.Iversen@rr-research.no (T.G.I.); Maria.Lyngaas.Torgersen@rr-research.no (M.L.T.); ksandvig@ibv.uio.no (K.S.); 2 Department of Molecular Cell Biology, Institute for Cancer Research, The Norwegian Radium Hospital, Oslo University Hospital, Montebello, 0379 Oslo, Norway; 3 Department of Biosciences, Faculty of Mathematics and Natural Sciences, University of Oslo, 0379 Oslo, Norway

**Keywords:** cell-penetrating peptides, cellular uptake, endocytosis, drug delivery, *in vivo* dose, drug delivery, pharmaceutical development

## Abstract

In this review, we discuss how cell-penetrating peptides (CPPs) might get access to their intracellular targets. We specifically focus on the challenge of deciding whether the positively-charged CPPs are just bound to the negatively-charged cell surface and subsequently endocytosed or actually transported into the cytosol, either by direct plasma membrane penetration or after endocytosis. This discussion includes comments about pitfalls when using pharmacological inhibitors in such studies. The possibility of exploiting CPPs as carriers for the delivery of drugs of different sizes *in vitro* is discussed, as is the use of CPPs as carriers for therapeutic drugs or contrast agents *in vivo*. We conclude that in many cases, more studies are needed to demonstrate conclusively whether increased delivery of a substance attached to CPPs is due to a membrane-penetrating property or whether the increase is a consequence of just changing the charge of the substance to be delivered. Finally, the expected dose needed for the use of such conjugates *in vivo* is discussed, including aspects to consider in order to bring potential products into clinical use.

## 1. Introduction

The therapy for several diseases, including cancer, might be improved by efficient delivery of various molecules into the cytosol and the nucleus of cells. Although small hydrophobic molecules are known to penetrate cell membranes, a number of drugs with cytosolic targets and most proteins and molecules, such as RNA and DNA, can neither penetrate cell membranes directly nor reach the cytosol after being endocytosed to any significant extent. Importantly, reports stating that small positively-charged peptides, such as the HIV1 TAT peptide, are membrane permeable initiated a number of studies to evaluate whether such cell-penetrating peptides (CPPs) could be used for drug delivery *in vitro* and *in vivo*. These peptides normally contain 5–30 amino acids. It is established that most CPPs have a high content of the positively-charged amino acid arginine, where the guanidinium group seems to be important for the properties of these peptides. Furthermore, the positively-charged amino acid lysine and various hydrophobic amino acids are often found in these peptides. For recent reviews, including the history, description and classification of different types of such peptides, as well as possibilities and challenges concerning their use, see [1,2,3,4].

Soon after the first report of CPPs, it became clear that many challenges had to be overcome in order to use these peptides in a successful strategy for drug delivery, at least *in vivo*. In the present article, we discuss the mechanisms for cellular entry of CPPs, as well as possibilities and challenges of using CPPs for the delivery of drugs *in vitro* and *in vivo*. We have also included some comments about issues that are important to consider in order to bring such products into clinical use. The views presented in the present article are based on the authors’ long experience with research related to cellular uptake and intracellular transport, as well as with *in vivo* drug delivery.

## 2. Entry Mechanisms for CPPs

The mechanisms of the intracellular entry of CPPs have been an issue for many discussions since these peptides were first reported to be membrane penetrating [1,2,4,5]. At least some of the early published conclusions about the membrane-penetrating properties of such peptides seem to be based on artefacts obtained during cell fixation after binding of the positively-charged peptides to the negatively-charged cell surface [4,6]. Later on, several groups published that the largest fraction of such peptides was transported into cells by endocytosis and, therefore, enters into intracellular vesicles in the endosomal-lysosomal pathway [7,8,9]. Thus, these peptides must be released or escape from endosomes into the cytosol, to avoid being exclusively transported to lysosomes, where they will be degraded.

Several reports indicate that at least some positively-charged peptides are able to escape from endosomes into the cytosol or directly penetrate the plasma membrane, at least when present at a concentration of 10 µM or above [4,8,10,11,12]. One should note that the intracellular localization of CPPs varies between different cell lines [12]. Thus, it looks like some of the substances now called CPPs are able to penetrate the plasma membrane directly and some are probably not, although researchers originally thought them to do so. Discriminating between cellular binding and uptake by endocytosis or penetration directly through the plasma membrane is not necessarily a simple issue and is further discussed below. We believe it is important for the future development of this field that the research community changes the nomenclature and uses the terminology CPPs or membrane-penetrating peptides for substances that without doubt have been shown to be membrane penetrating. As it now stands, it looks like the terminology CPP is used also for peptides that just bind to the negatively-charged cell surface and then are taken up by endocytosis. However, in this article, we have chosen to continue using the terminology CPPs as it has been used up to now.

## 3. Binding of CPPs to Cells and Uptake via Endocytosis

All positively-charged molecules would be expected to bind to the cell surface, which has a net negative charge, mainly due to the presence of glycoproteins (e.g., glycosaminoglycans, like heparan sulfate) and glycolipids, such as gangliosides (which contain negatively-charged sialic acid). Several positively-charged CPPs bind to the negatively-charged glycosaminoglycans on the cell surface [4,10,13]. Substances binding to the cell surface can be expected to enter cells by at least one of the many endocytic mechanisms [14,15,16]. Such uptake mechanisms are highly active. A macrophage may ingest the whole plasma membrane in approximately 30 min and fibroblasts in 2 h (this corresponds to 20%–25% of the cell volume in macrophages and 5%–10% of the total cell volume in fibroblasts per hour) [17]. These processes are important for, e.g., the uptake of nutrients, downregulation of growth factor receptors and regulation of many cellular functions. Even substances just being in the neighborhood of cells (without being bound to the cells) may enter into cells via endocytosis, then often referred to as pinocytosis or cell drinking [16].

**Figure 1:**
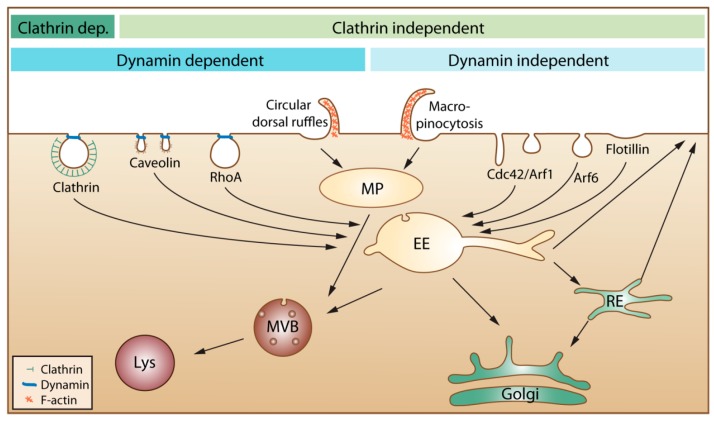
An overview of cellular endocytic mechanisms. The various endocytic uptake mechanisms are presented according to their dependency on either clathrin or dynamin. Most internalized cargo ends up in a common compartment, the early endosome (EE). From EEs, the cargo can either be directly recycled to the plasma membrane or be sorted to the recycling endosome (RE) before returning to the cell surface. Cargo that is not recycled may move down the endo-lysosomal pathway via multivesicular bodies (MVB) for degradation in the lysosome (Lys). Clathrin dep: Clathrin dependent. MP: macropinosome.

In the present article, we do not aim at providing a detailed description of different endocytic mechanisms; we instead refer to recent review articles by us and others for more information [16,18,19,20,21]. In the published articles, one can often see that the authors describe endocytosis as mainly being due to clathrin-mediated endocytosis, caveolae-mediated endocytosis and macropinocytosis. We would like to mention that several other mechanisms are involved, as illustrated in Figure 1.

Moreover, the content of all endocytic vesicles (possibly with the exception of macropinosomes) ends up in the early endosomes before being transported further into the cell (Figure 1). It is also important to be aware that the amount of caveolae varies greatly between different tissues and individual cells and that caveolae are concentrated on the basolateral surface of epithelial cells [22]. Thus, it is presently not clear how much caveolae contribute to uptake in most cell types. Moreover, caveolae that pinch off were earlier thought to form vesicles called caveosomes. These structures were reported to have a neutral pH, and their content was published to go directly to the endoplasmic reticulum and avoid lysosomal degradation. The caveosome has now been shown to be an artefact due to overexpression of caveolin-1 [23]. Thus, as suggested by the authors who first published this structure, the term caveosome should not be used anymore [23], and articles describing uptake via caveosomes should be viewed in light of this new knowledge.

**Figure 2:**
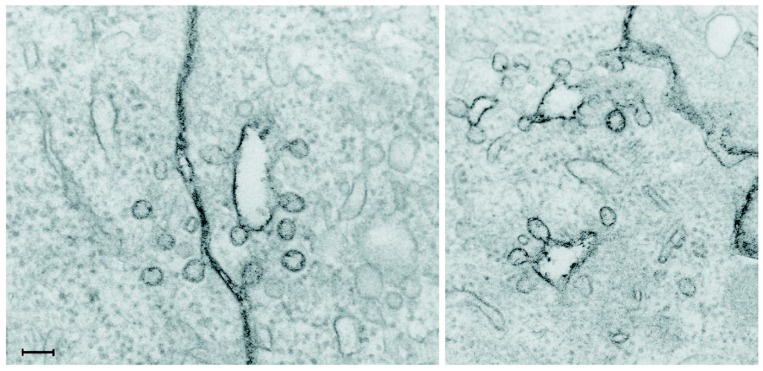
Electron microscopy image taken after staining of the plasma membrane with ruthenium red added during the fixation. Some of the structures revealed by this staining to be plasma membrane connected might otherwise have been interpreted as being intracellular vesicles. The scale bar is 100 nm. Reproduced from [18].

Studying whether a substance is just bound to cells or taken up into cells by endocytosis is not straight forward. A diffuse staining throughout the cell is often interpreted as a cytosolic localization, but could in fact also be due to cell surface staining if not properly controlled. We have a few years ago discussed the challenges and pitfalls in studying cellular uptake of nanoparticles [16]. The discussion in that article still holds true, also for studies with CPPs. This includes judging whether a substance is just bound to the cell surface or taken up into the cell, which can be difficult due to the many invaginations, ruffles and other irregular structures on the cell surface. This is illustrated in Figure 2, which shows an image taken by electron microscopy after staining of the plasma membrane with ruthenium red added during the fixation, implying that all stained areas are surface connected [18]. Clearly, some of the structures revealed by ruthenium red staining to be plasma membrane connected might easily have been interpreted as being intracellular vesicles, since they appear to be inside the cell.

Fluorescently-labelled molecules are undoubtedly very useful to study endocytic uptake, but adding such labels to relatively small peptides may drastically change the ability of the substance to interact with or be taken up by cells. Moreover, it is not straight forward to determine if the fluorescent group is bound to the surface or if it is present inside the cell, partly due to the low resolution of light microscopy and partly due to irregular membranes on cells, which also may be quite flat, such as endothelial cells. To determine if a substance is taken up into cells by endocytosis, it is common to include different pharmacological inhibitors of endocytosis or to use the knock-down of proteins known to be involved in this process. When it comes to possible pitfalls with such studies, we refer to our earlier review, which includes a toolbox for the use of pharmacological inhibitors [16]. It should be mentioned that also flow cytometry does not discriminate between substances just bound to cells or being internalized. The use of non-membrane permeable fluorescence quenchers might be a useful tool to see if a fluorescent group is bound outside the cell or taken up into the cell. One should then, however, be careful to use very small quenchers and check if a reduced fluorescence is not just due to the quencher not being able to come close enough to the fluorescent group (compare our discussion about the many small invaginations on the cell surface discussed above). Moreover, it might be considered if surface bound CPPs can be removed by proteases. Substances taken up into cells by endocytosis will first be localized in the small intracellular vesicles called endosomes. From these endosomes, they may be transported through the endosomal pathway to lysosomes (Figure 1), which contain many hydrolases and proteases and, thus, are cleaving most substances reaching these vesicles. Inside early endosomes, the pH is 6.0–6.5, and it drops further as the endosomes mature and become late endosomes, which fuse with lysosomes (pH 4.5–5.5). This low pH, caused by an ATP-driven proton pump, may in the presence of high amounts of, e.g., imidazole-containing substances or polymers, such as polyethylenimine, help substances to escape from the vesicles by the so-called proton-sponge effect, *i.e*., vesicle swelling and disruption due to increased osmotic pressure. These substances will, due to their buffering capacity, increase the influx of H^+^, the counter ion Cl^−^ and H_2_O [24]. More studies are, however, needed to explore how efficient and useful this endosomal escape mechanism can be, especially *in vivo*.

### 3.1. In Vitro Drug Delivery Using CPPs

Studies performed with cells grown in culture have clearly demonstrated that CPPs can enter the cytosol, either directly through the plasma membrane or after endocytic uptake [4,5]. As discussed above, the addition of positively-charged substances would be expected to mediate cellular uptake, at least by endocytosis, and, for some substances, by penetration through the plasma membrane. An important issue regarding drug delivery to the cytosol using CPPs is how large a substance can be and which other properties it can have, without disrupting the membrane-penetrating ability of the CPP. One can expect the membrane-penetrating property to depend on the size of the CPP-coupled substance and to decrease for CPPs coupled to large molecules or particles [4,11]. Thus, one should be extra cautious when interpreting data obtained for cellular uptake with large substances bound to membrane-penetrating CPPs, in judging whether also the complex is membrane penetrating, just bound to the surface or perhaps exclusively taken up by endocytosis. Notably, it was recently reported that a rapid cytosolic delivery was obtained with cyclic TAT peptides coupled to green fluorescent protein and that the cyclic peptide resulted in higher transduction efficiency than using the non-cyclic peptide [25]. It will be interesting to see if the strategy of making cyclic peptides also improves the delivery of other CPPs.

Several authors have reported coupling of CPPs to nanoparticles in order to bring the particles into cells, e.g., [26,27], and although different conclusions have been reached, most particles seem to end up in lysosomes [28]. Even in a case where 16-nm gold nanoparticles coupled to a positively-charged CPP were reported to be observed in the cytosol [29], the fraction remaining in endosomes was close to 100% (see the discussions in [16,30]). It might, however, be sufficient for therapeutic purposes, as well as studies in basic cell biology that a minor fraction is able to reach the cytosol, and endosomal conditions may facilitate this transfer. Regarding such discussions, we would like to draw attention to a study with lipid nanoparticles not containing CPPs [31]. In that article, the cellular distribution of 60-nm lipid nanoparticles loaded with siRNA and labelled with 6-nm gold particles was carefully quantified. The authors concluded that only 1%–2% of the gold particles were able to escape from endosomes to the cytosol, and this escape only happened from a compartment sharing early and late endosomal characteristics. In spite of this low efficiency in transfer to the cytosol, the siRNA downregulated 90% of its target.

One should keep in mind that when the authors claim that they have succeeded in delivering a substance to cytosol, that delivery may be quite inefficient, although detectable when using a sensitive assay. It has, for example, been shown that only one molecule of some toxins is sufficient to kill a cell [32,33], and only one RNA/DNA molecule encoding a fluorescent probe or a toxic substance might be detected.

### 3.2. In Vivo Drug Delivery Using CPPs

Clinical studies with CPP-based products have shown promise for topical delivery of drugs [2]. However, we are not aware of any studies demonstrating CPPs being able to deliver drugs effectively into cells following intravenous injection. Since most CPPs are positively charged or rather hydrophobic, one would expect them to bind to blood plasma proteins and several types of cells in the body. In general, such protein binding will contribute to longer circulation times, whereas binding to different cells will contribute to an unspecific biodistribution [30]. Furthermore, non-selective membrane penetration of CPPs should be expected to cause a wide biodistribution and drug exposure of healthy tissue.

CPPs cannot be expected to bind selectively to disease-specific structures. An important question is therefore: will it be possible to improve the biodistribution and selectivity for diseased areas by adding targeting molecules to the CPPs? If one adds a targeting molecule to increase the binding to disease specific structures, will the CPPs still be able to bring the substance into the cytosol, or will it merely be taken up by endocytosis? If CPPs in such a case do not improve penetration to the cytosol, it only makes the product more complicated than necessary. It is important to keep in mind that in order to get new products approved for clinical use, it is, as discussed below, important to make the products as simple as possible [34]. As follows from this discussion, there will obviously be many questions and challenges that have to be solved in making use of the membrane-penetrating properties of CPPs to deliver drugs following intravenous injection.

One important issue to take into consideration when discussing drug delivery and biodistribution data obtained with CPPs or CPP-conjugates is to what extent a difference in the biodistribution of a substance and the CPP-conjugate of that substance is due to the membrane-penetrating properties or whether it is just an effect of changing the charge of the substance. Many published results demonstrating improved drug delivery for large molecules or nanoparticles after binding of CPPs may just be due to changing the charge of the injected substance. Control experiments where, e.g., single, positively-charged, amino acids instead of CPPs are added to the substance to be delivered are almost never reported. Of course, the most important issue for drug delivery is whether it works. Our point is just to stress that an observed drug delivery of a substance coupled to CPPs is not necessarily evidence for a membrane-penetrating property; it might just be an effect of a charge-induced change of biodistribution. We believe that in order to bring research with CPPs forward as fast as possible, it is important that one in the scientific literature does not define a peptide as a CPP with a membrane-penetrating effect without demonstrating such an effect.

Many researchers focus on the possibility to use CPPs to deliver contrast agents for medical imaging. The goal of such molecular imaging is to visualize and quantify biomarkers or biochemical and cellular processes with high sensitivity, specificity and signal-to-noise ratios for early detection of disease, identifying the extent of disease, selecting treatment and measuring the effect of treatment [35,36]. We believe that the use of CPPs for such purposes will be even more challenging than the use of CPPs for the delivery of therapeutics. The reason for this statement is that the most important issue when making contrast agents for medical imaging is to obtain a good signal-to-background level, which makes it very important to have a rapid excretion of the agents from areas close to the diseased area [30]. There is a hypothetical possibility that a diseased cell is more permeable to the peptide than the neighboring cells, but so far, there are no examples of such selectivity. Another scenario is more likely: the membrane-penetrating imaging agent is able to reach a target in the cytosol; but one would then expect that to happen also for neighboring cells, and it would be a challenge to reduce the signal from cells just outside the diseased area. Furthermore, due to the expected slow excretion of contrast agents from neighboring cells, the strategy of using CPPs to deliver contrast agents would be expected to be useful only in cases where both the disease to image and the modality used allow imaging many hours after injection of the agent. This approach is therefore probably not useful for positron emission tomography (PET), where the most commonly-used isotope ^18^F has a half-life of 110 min, and may even be restricted for the use of single photon emission computer tomography (SPECT), where the most commonly-used isotope ^99m^Tc has a half-life of six hours [30]. Another argument against using CPPs for imaging purposes is that for imaging, it is sufficient that the agent binds to and gives a good signal-to-background signal in the diseased area; it is not necessary that the agent enters into cells.

Finally, we give some words on the expected dose needed for intravenously-injected CPPs. Reports in the literature indicate that a dose of at least 10 µM of the CPPs may be necessary in order to make the CPPs membrane-penetrating and reach the cytosol at conditions when all known forms of endocytosis are knocked down [4,12]. For a peptide containing 10 amino acids, a concentration of 10 µM means approximately 10 µg/mL. Approximately 30 mg of substance have to be injected in order to obtain a concentration of 10 µM of such a substance in a volume similar to that of the blood plasma (3000 mL). Much higher amounts have to be injected to obtain concentrations above 10 µM for more than a few seconds in blood and reach such concentrations in the diseased tissues. Thus, the dose needed to obtain the membrane-penetrating properties for such peptides is rather high, especially when compared to the expected human dose of 50–100 µg for two targeting peptides for SPECT imaging, with which one of the authors has been working [37,38].

## 4. Development for Clinical Use

Positively-charged substances, like CPPs, are expected to give a wide biodistribution following intravenous injection and to be slowly excreted from the body. For substances staying in the body for a long time, toxicity studies have to last longer, and the cost of the development studies is therefore higher than for substances being more rapidly excreted [34,39]. A distribution with as much as possible of the injected dose homing to the target is very important, both to obtain the wanted effect (therapeutic or imaging) and to reduce unwanted effects. The safety issues of injecting such compounds and to look for an effect on non-targeted cells have not been investigated sufficiently so far; obviously, these aspects deserve more focus in future studies. If the local concentration of CPPs needs to be 10 µM or higher to obtain a direct membrane-penetrating effect, the injected dose has to be high, which is important for the cost of such drugs. Last, but not least, it is important to take into consideration that not only does a new drug need to be documented to function, but it also has to be better or less expensive than competing products. We have in a recent article discussed what is needed to bring nanoparticles to clinical use [34]. That discussion includes how the complexity of drugs affects the cost of drug development. It also includes discussions about the risk/benefit evaluations one can expect pharmaceutical companies to perform before starting large development projects. All issues discussed in that article are also relevant for what is needed to bring CPP-based products into clinical use.

## 5. Concluding Remarks

In this article, we have discussed the challenges in determining whether CPPs are membrane penetrating or whether they are just taken up by endocytosis. If taken up by endocytosis, the substance needs to escape endosomal vesicles to reach targets in other organelles or the cytosol. The examples discussed show, however, that important biological effects might be obtained even if the substance released from endosomes may be only a small percentage of the substance endocytosed. We conclude that the possibility of benefitting from using CPPs for drug delivery is largest for *in vitro* use and that it is more likely that one will be able to successfully use CPPs to deliver therapeutics than to deliver imaging agents.
